# Risk assessment of assisted reproductive technology and parental age at childbirth for the development of uniparental disomy-mediated imprinting disorders caused by aneuploid gametes

**DOI:** 10.1186/s13148-023-01494-w

**Published:** 2023-05-06

**Authors:** Kaori Hara-Isono, Keiko Matsubara, Akie Nakamura, Shinichiro Sano, Takanobu Inoue, Sayaka Kawashima, Tomoko Fuke, Kazuki Yamazawa, Maki Fukami, Tsutomu Ogata, Masayo Kagami

**Affiliations:** 1 Department of Molecular Endocrinology, National Research Institute for Child Health and Development, 2-10-1 Okura, Setagaya-Ku, Tokyo, 157-8535 Japan; 2 Department of Pediatrics, Keio University School of Medicine, 35 Shinanomachi, Shinjuku-Ku, Tokyo, 160-8582 Japan; 3 Department of Pediatrics, Hokkaido University Graduate School of Medicine, Kita 15, Nishi 7, Kita-Ku, Sapporo, 060-8648 Japan; 4 Department of Endocrinology and Metabolism, Shizuoka Children’s Hospital, 860 Urushiyama, Aoi-Ku, Shizuoka, 420-8660 Japan; 5 Medical Genetics Center, National Hospital Organization Tokyo Medical Center, 2-5-1 Higashigaoka, Meguro-Ku, Tokyo, 152-8902 Japan; 6 Department of Biochemistry, Hamamatsu University School of Medicine, 1‑20‑1 Handayama, Higashi‑ku, Hamamatsu, 431‑3192 Japan; 7 Department of Pediatrics, Hamamatsu Medical Center, 328 Tomizuka Cho, Naka-Ku, Hamamatsu, 432-8580 Japan

**Keywords:** Assisted reproductive technology, Imprinting disorders, Uniparental disomy, Maternal age, Risk factors

## Abstract

**Background:**

Our previous study suggested that assisted reproductive technology (ART) may be a possible risk factor for the development of epimutation-mediated imprinting disorders (epi-IDs) for mothers aged ≥ 30 years. However, whether ART or advanced parental age facilitates the development of uniparental disomy-mediated IDs (UPD-IDs) has not yet been investigated.

**Results:**

We enrolled 130 patients with aneuploid UPD-IDs including various IDs confirmed by molecular studies and obtained ART data of the general population and patients with epi-IDs from a robust nationwide database and our previous report, respectively. We compared the proportion of ART-conceived livebirths and maternal childbearing age between patients with UPD-IDs and the general population or patients with epi-IDs. The proportion of ART-conceived livebirths in patients with aneuploid UPD-IDs was consistent with that in the general population of maternal age ≥ 30 years and was lower than that in the patients with epi-IDs, although there was no significant difference. The maternal childbearing age of patients with aneuploid UPD-IDs was skewed to the increased ages with several cases exceeding the 97.5th percentile of maternal childbearing age of the general population and significantly higher than that of patients with epi-IDs (*P* < 0.001). In addition, we compared the proportion of ART-conceived livebirths and parental age at childbirth between patients with UPD-IDs caused by aneuploid oocytes (oUPD-IDs) and that by aneuploid sperm (sUPD-IDs). Almost all ART-conceived livebirths were identified in patients with oUPD-IDs, and both maternal age and paternal age at childbirth were significantly higher in patients with oUPD-IDs than in patients with sUPD-IDs. Because maternal age and paternal age were strongly correlated (*r*_*s*_ = 0.637, *P* < 0.001), higher paternal age in oUPD-IDs was explained by the higher maternal age in this group.

**Conclusions:**

Different from the case of epi-IDs, ART itself is not likely to facilitate the development of aneuploid UPD-IDs. We demonstrated that advanced maternal age can be a risk factor for the development of aneuploid UPD-IDs, particularly oUPD-IDs.

**Supplementary Information:**

The online version contains supplementary material available at 10.1186/s13148-023-01494-w.

## Background

Uniparental disomy (UPD) is defined as a condition in which both homologs of a chromosome are inherited from only one parent [1]. Maternal UPD (UPDmat) and paternal UPD (UPDpat) consist of two homologs derived from only the mother and only the father, respectively. Isodisomy refers to the inheritance of duplicated identical homologs from one parent, whereas heterodisomy refers to the inheritance of both non-identical homologs from one parent. UPD is caused by four mechanisms, namely trisomy rescue (TR), gamete complementation (GC), monosomy rescue (MR), and post-fertilization mitotic error (PE) (Additional file 1: Fig. S1) [1]. TR-type and GC-type UPDs show heterodisomy, and MR-type UPD shows full isodisomy. PE-type UPD includes mosaic full isodisomy with normal cell lineage and segmental isodisomy. In particular, PE with a ring chromosome results in full isodisomy with normal cell lineage, through loss of a ring chromosome followed by duplication of a normal chromosome [2, 3]. As shown in Additional file 1: Fig. S1, TR, GC, and MR types of UPDs are caused by aneuploid sperm or oocytes (aneuploid UPDs). Briefly, TR-type and GC-type UPDs are mediated by disomic oocytes in UPDmat and disomic sperm in UPDpat (Additional file 1: Fig. S1). MR-type UPD is mediated by nullisomic oocytes in UPDpat and nullisomic sperm in UPDmat (Additional file 1: Fig. S1). In this regard, TR-type and GC-type UPDmat and MR-type UPDpat are considered as aneuploid oocyte-mediated UPDs, whereas TR-type and GC-type UPDpat and MR-type UPDmat are considered as aneuploid sperm-mediated UPDs.

Imprinting disorders (IDs) are clinical syndromes caused by abnormal expression of the imprinted genes, which express in parental origin specific manner [4]. The etiologies of IDs include pathogenic variants in causative genes, structural abnormalities affecting the imprinted regions, UPD of chromosomes having imprinted genes, and aberrant methylation of the disease-responsible differential methylated regions (DMRs), i.e., epimutation [4]. The relative frequency of UPD differs among IDs (Additional file 2: Table S1). UPD is the most frequent genetic cause of several IDs, such as transient neonatal diabetes mellitus caused by UPDpat of chromosome 6, Temple syndrome caused by UPDmat of chromosome 14 (UPD(14)mat), and Kagami-Ogata syndrome (KOS) caused by UPDpat of chromosome 14 (UPD(14)pat) (Additional file 2: Table S1).

Advanced maternal age at childbirth (≥ 35 years) is known to be a risk factor for the development of aneuploid oocytes due to chromosome segregation errors during meiosis, in particular, meiosis 1 (M1) [5, 6]. Consistent with this, several studies have shown advanced maternal age in patients with TR-type or GC-type UPDmat [7, 8] and MR-type UPD(14)pat [9] mediated by disomic and nullisomic oocytes, respectively. In addition, Nakka et al. reported that mothers of patients with UPDmat were significantly older than those of non-UPD individuals, based on the database consisting of four million individuals from the general population [10]. However, the effect of advanced paternal age on the development of aneuploid sperm and UPDpat remains to be elucidated.

Assisted reproductive technology (ART), including controlled ovarian stimulations (COS), in vitro maturation and cryopreservation of oocytes, in vitro fertilization (IVF), intracytoplasmic sperm injection (ICSI), embryo culture, and embryo transfer, may affect the epigenetic modification at the imprinted region during gametogenesis and embryonic development in the preimplantation stage [11]. Therefore, ART has been considered as a risk factor for the development of IDs, particularly epimutation-mediated IDs (epi-IDs). Several studies, including IDs with all genetic causes (UPD, structural abnormalities, epimutation, and variants in causative genes), showed that the frequency of ART-conceived livebirths was higher in patients with Beckwith-Wiedemann syndrome (BWS), Silver-Russell syndrome (SRS), Angelman syndrome (AS), and Prader-Willi syndrome (PWS) than in the general population [12, 13]. In our previous study focused on epi-IDs, we demonstrated that ART can be a risk factor for the development of epi-IDs, particularly BWS and SRS, in mothers aged over 30 years [14]. However, it remains controversial whether ART increases the risk for the development of aneuploid gametes and aneuploid UPD in zygotes. COS, particularly using high-dose gonadotropins, has been reported to increase the frequency of oocyte aneuploidy because of stimulated meiotic progression that leads to segregation errors [15, 16]. On the contrary, a recent retrospective study in Chinese women using anonymized data on preimplantation genetic screening for blastocysts found that gonadotropin dosage is not associated with embryonic aneuploidy [17]. In addition, molecular cytogenic analysis of early spontaneous abortions revealed that IVF and ICSI did not enhance aneuploidy rate [18]. Although a previous study assessed the risk of ART focusing on PWS due to UPDmat of chromosome 15 (UPD(15)mat) [19], there was no study which evaluated the effect of ART on the development of various aneuploid UPD-mediated IDs (UPD-IDs). Moreover, the confounding effect of advanced parental age at childbirth remains to be elucidated.

To clarify whether ART or advanced parental age at childbirth facilitates the development of aneuploid UPD-IDs, we compared (1) the proportion of ART-conceived livebirths and the distribution of maternal childbearing age between patients with aneuploid UPD-IDs and that of the general population or patients with epi-IDs by utilizing previous data from our cross-sectional study [14], and (2) the proportion of ART-conceived livebirths and parental age at childbirth between patients with UPD-IDs caused by aneuploid oocytes and those with UPD-IDs caused by aneuploid sperm.

## Results

### Numbers of patients with aneuploid UPD-IDs

We enrolled 130 patients with aneuploid UPD-IDs confirmed by molecular analyses as shown in Methods and obtained clinical information about parental age at childbirth, conception (naturally or ART-conceived), and ART methods utilized in ART-conceived patients. As in our previous report [14], we classified patients conceived with IVF, ICSI, and FET (frozen embryo transfer) into ART-conceived livebirths based on the definition used by the Japanese Society of Obstetrics and Gynecology (JSOG). Patients born after COS only were not included in ART-conceived livebirths. Of 130 patients with aneuploid UPD-IDs, information about conception, paternal age, and maternal age at childbirth was obtained from 122, 125, and 130 patients, respectively. The numbers of patients with aneuploid UPD-IDs and affected chromosomes are summarized in Table 1. A large number of patients with SRS caused by UPDmat of chromosome 7 (UPD(7)mat), KOS caused by UPD(14)pat, and PWS caused by UPD(15)mat were included. We classified disomic oocyte-mediated matUPDs (caused by TR or GC) and nullisomic oocyte-mediated patUPDs (caused by MR) into UPD-IDs caused by aneuploid oocytes, and disomic sperm-mediated patUPDs (caused by TR or GC) and nullisomic sperm-mediated matUPDs (caused by MR) into UPD-IDs caused by aneuploid sperm, based on the UPD subtypes confirmed by microsatellite analysis and SNP array analysis. We identified 104 and 26 UPD-IDs caused by aneuploid oocytes and sperm, respectively (Table 1).

**Table 1:** Summary of patients with aneuploid UPD-IDs in this study

Chromosome	IDs associated with maternal UPD	Number of patients	IDs associated with paternal UPD	Number of patients
6	UPD(6)mat	2	UPD(6)pat	1
		(Hetero: 2)	Transient neonatal diabetes mellitus	(Iso: 1)
7	UPD(7)mat	25^a^	UPD(7)pat	1
	Silver-Russell syndrome	(Hetero: 17, Iso: 8)		(Iso: 1)
14	UPD(14)mat	18^b^	UPD(14)pat	27^c^
	Temple syndrome	(Hetero: 15, Iso: 3)	Kagami-Ogata syndrome	(Hetero: 10, Iso: 17)
15	UPD(15)mat	48^d^	UPD(15)pat	2
	Prader-Willi syndrome	(Hetero: 44, Iso: 4)	Angelman syndrome	(Iso: 2)
20	UPD(20)mat	6^e^	UPD(20)pat	0
		(Hetero: 5, Iso: 1)	Pseudo hypoparathyroidism 1B	
Total		Hetero: 83, Iso: 16		Hetero: 10, Iso: 21

	Maternal UPD subtype		Paternal UPD subtype		Total
UPD-IDs caused by aneuploid oocytes	heterodisomy	83	isodisomy	21	104
UPD-IDs caused by aneuploid sperms	isodisomy	16	heterodisomy	10	26

UPD, uniparental disomy; IDs, imprinting disorders; UPD-IDs, uniparental disomy-mediated imprinting disorders; Hetero, heterodisomy; Iso, isodisomy; UPD(6)mat, maternal uniparental disomy of chromosome 6; UPD(6)pat, paternal uniparental disomy of chromosome 6; UPD(7)mat, maternal uniparental disomy of chromosome 7; UPD(7)pat, paternal uniparental disomy of chromosome 7; UPD(14)mat, maternal uniparental disomy of chromosome 14; UPD(14)pat, paternal uniparental disomy of chromosome 14; UPD(15)mat, maternal uniparental disomy of chromosome 15; UPD(15)pat, paternal uniparental disomy of chromosome 15; UPD(20)mat, maternal uniparental disomy of chromosome 20; UPD(20)pat, paternal uniparental disomy of chromosome 20

^a^9 out of 25 UPD(7)mat patients were reported by Fuke et al. [20]

^b^17 out of 18 UPD(14)mat patients were reported by Kagami et al. [22]

^c^19 out of 27 UPD(14)pat patients were reported by Kagami et al. [21]

^d^27 out of 48 UPD(15)mat patients were reported by Matsubara et al. [17]

^e^5 out of 6 UPD(20)mat patients were reported by Kawashima et al. [23]

### Comparison of the proportion of ART-conceived livebirths and maternal childbearing age between patients with aneuploid UPD-IDs and the general population or patients with epi-IDs

**Fig. 1:**
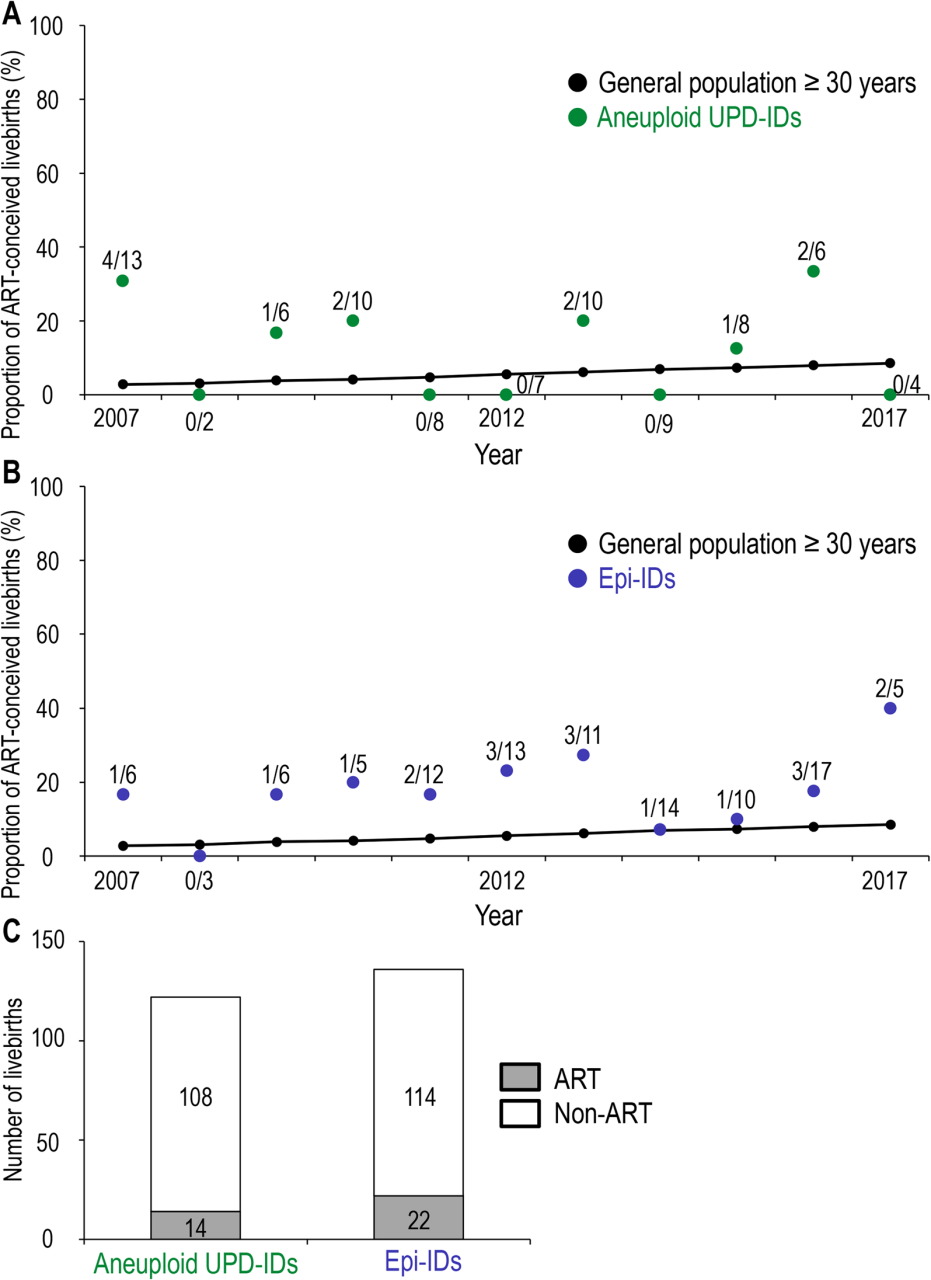
Comparison of the proportion of ART-conceived livebirths. **A** The proportion of ART-conceived livebirths in patients with aneuploid UPD-IDs and that in the general population between 2007 and 2017. Green dots indicate the proportion of ART-conceived livebirths in patients with aneuploid UPD-IDs with actual numbers (ART-conceived livebirths/total livebirths). **B** The proportion of ART-conceived livebirths in patients with epi-IDs and that in the general population between 2007 and 2017, cited as Fig. 2B in reference #14. Blue dots indicate the proportion of ART-conceived livebirths in patients with epi-IDs with actual numbers (ART-conceived livebirths/total livebirths). **C** The proportion of ART-conceived livebirths in patients with aneuploid UPD-IDs and epi-IDs. Gray and white bars indicate the number of ART-conceived and non-ART-conceived livebirths, respectively. ART, assisted reproductive technology; UPD-IDs, uniparental disomy-mediated imprinting disorders; epi-IDs, epimutation-mediated imprinting disorders

To clarify whether ART and advanced maternal childbearing age bear the risk for the development of aneuploid UPD-IDs, we compared (1) the proportion of ART-conceived livebirths and (2) the distribution of the maternal childbearing age, between patients with aneuploid UPD-IDs and the general population or patients with epi-IDs. The data of the general population and patients with epi-IDs were obtained from our previous report [14]. Of 122 patients with aneuploid UPD-IDs whose information about conception was available, 14 (11.5%) were conceived with ART. Figure 1 shows the comparison of the proportion of ART-conceived livebirths in the patients with aneuploid UPD-IDs (Fig. 1A) or epi-IDs (Fig. 1B) and that in the general population, every year from 2007 to 2017. Because we previously demonstrated that the proportion of mothers aged ≥ 30 years was more than 90% in ART pregnancy in Japan [14], we compared the proportion of ART-conceived livebirths in all patients with aneuploid UPD-IDs and that in the general population of childbearing age ≥ 30 years from 2007 to 2017 when we could obtain the age distribution of mothers who conceived with ART from the JSOG database. The proportion of ART-conceived livebirths in the patients with aneuploid UPD-IDs was generally consistent with that in the general population of maternal age ≥ 30 years with some variation in each year (Fig. 1A). However, the proportion of ART-conceived livebirths in epi-IDs was higher than that in the general population (Fig. 1B). The comparison of the proportion of ART-conceived livebirths in the patients with aneuploid UPD-IDs and epi-IDs is shown in Fig. 1C. The proportion of ART-conceived livebirths in aneuploid UPD-IDs (11.4%) was lower than that in epi-IDs (16.2%); however, there was no significant difference (*P* = 0.288).

**Fig. 2:**
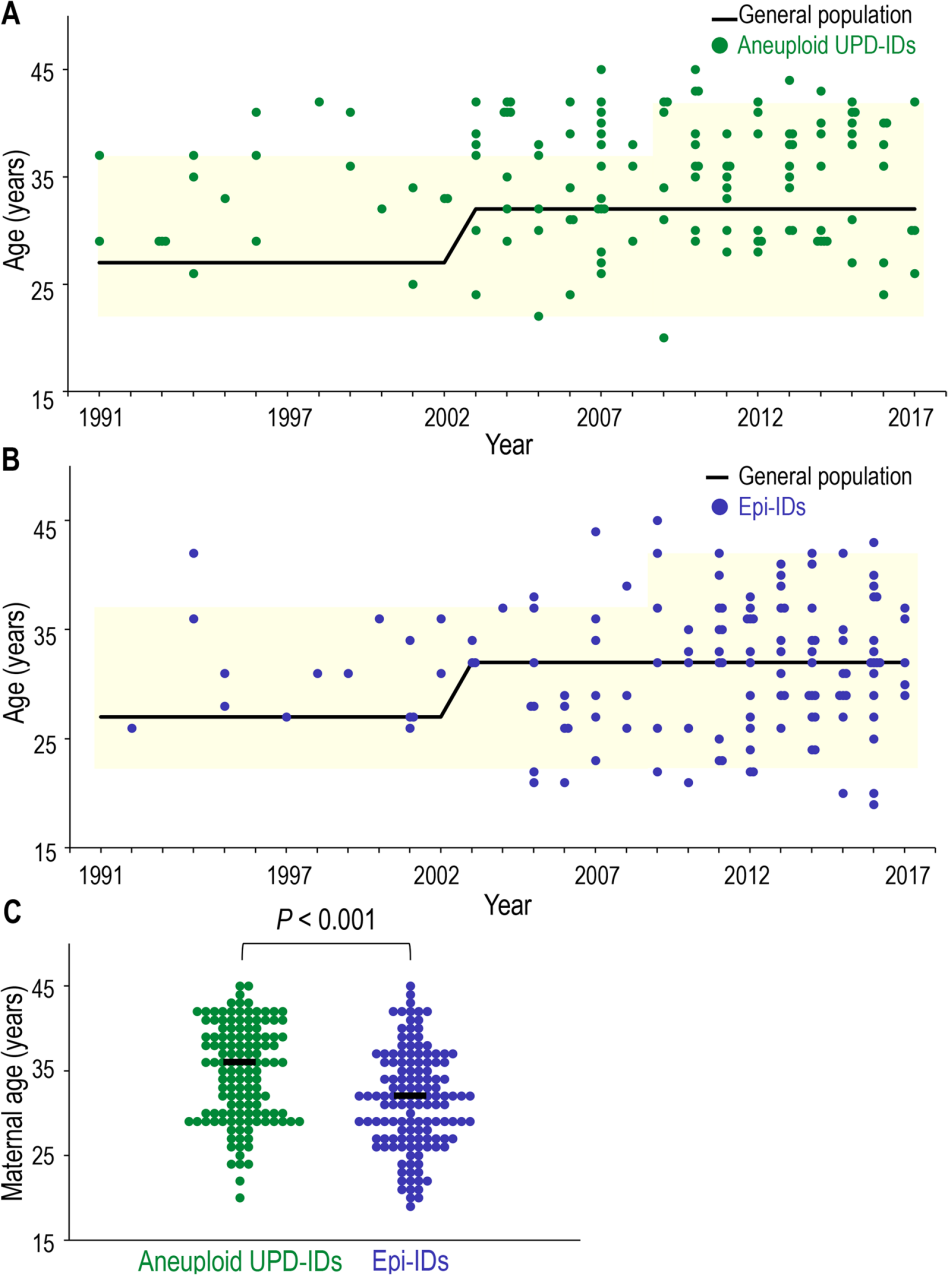
Comparison of maternal childbearing age. **A** The distribution of maternal childbearing age in the patients with aneuploid UPD-IDs between 1991 and 2017. Green dots indicate the maternal age of the patients with aneuploid UPD-IDs in each year. The black line indicates the median age of the general population and the yellow area indicates the 2.5th to 97.5th percentiles of the general population. **B** The distribution of maternal childbearing age in the patients with epi-IDs between 1991 and 2017, modified from Fig. 3 in reference #14. Blue dots indicate the maternal age of the patients with epi-IDs in each year. The black line indicates the median age of the general population and the yellow area indicates the 2.5th to 97.5th percentiles of the general population. **C** The distribution of maternal age in patients with aneuploid UPD-IDs and epi-IDs. Black bars indicate the median maternal age of patients in each group. UPD-IDs, uniparental disomy-mediated imprinting disorders; epi-IDs, epimutation-mediated imprinting disorders

The distribution of maternal childbearing age in patients with aneuploid UPD-IDs and epi-IDs between 1991 and 2017 is shown in Fig. 2. The distribution of maternal childbearing age of patients with aneuploid UPD-IDs was skewed toward the increased ages (median 36) with several cases exceeding the 97.5th percentile of maternal childbearing age of the general population (Fig. 2A), whereas that of the patients with epi-IDs varied widely from 19 to 45 (median 32) within the approximate 2.5th to 97.5th percentiles of maternal childbearing age of the general population (Fig. 2B). The comparison of maternal age between aneuploid UPD-IDs and epi-IDs showed that maternal age in aneuploid UPD-IDs was significantly higher than that in epi-IDs (*P* < 0.001) (Fig. 2C).

### Comparison of the proportion of ART-conceived livebirths and parental age at childbirth between patients with UPD-IDs caused by aneuploid oocytes and that with UPD-IDs caused by aneuploid sperm

**Fig. 3:**
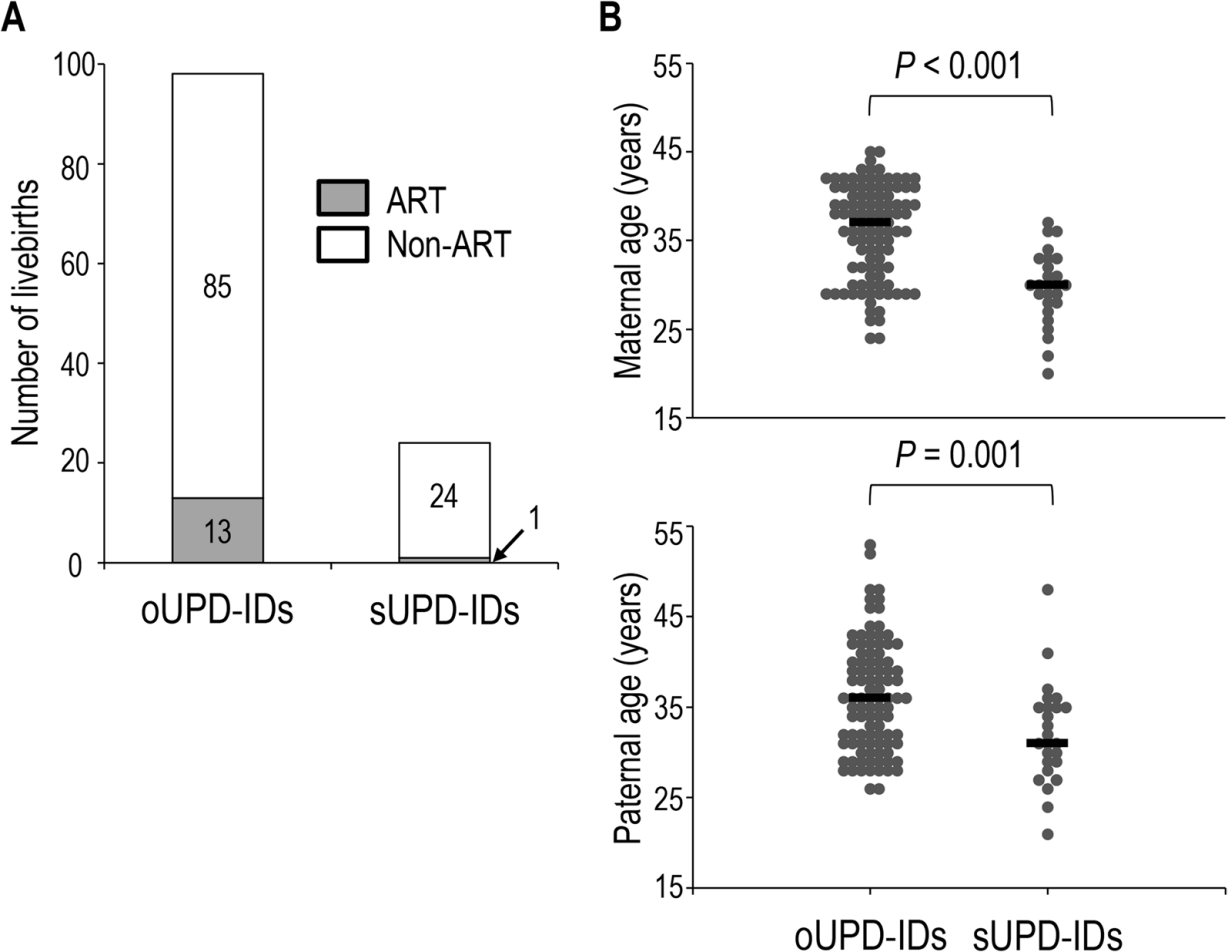
Comparison of the proportion of ART-conceived livebirths and the distribution of maternal age and paternal age at childbirth between patients with oUPD-IDs and sUPD-IDs **A** The proportion of ART-conceived livebirths in patients with oUPD-IDs and sUPD-IDs. Gray and white bars indicate the number of ART-conceived and non-ART-conceived livebirths, respectively. **B** The distribution of maternal and paternal age at childbirth in patients with oUPD-IDs and sUPD-IDs. Black bars indicate the median maternal or paternal age of patients in each group. ART, assisted reproductive technology; oUPD-IDs, uniparental disomy-mediated imprinting disorders caused by aneuploid oocytes; sUPD-IDs, uniparental disomy-mediated imprinting disorders caused by aneuploid sperm

To investigate whether ART and parental age had different effects for the gametes in the development of aneuploid UPD-IDs, we compared the proportion of ART-conceived livebirths and parental age at childbirth between patients with UPD-IDs caused by aneuploid oocytes and that with UPD-IDs caused by aneuploid sperm. The proportion of ART-conceived livebirths in UPD-IDs caused by aneuploid oocytes (oUPD-IDs) (13.3%) was higher than that in UPD-IDs caused by aneuploid sperm (sUPD-IDs) (4.0%) (Fig. 3A). Notably, almost all ART-conceived livebirths were identified in patients with oUPD-IDs. Both maternal age and paternal age at childbirth of patients with oUPD-IDs were higher than that of patients with sUPD-IDs (maternal: median 37 vs. 30, *P* < 0.001; paternal: median 36 vs. 31, *P* = 0.001) (Fig. 3B). In addition, both maternal age and paternal age at childbirth were strongly correlated in all patients with aneuploid UPD-IDs (*r*_*s*_ = 0.637, *P* < 0.001).

### Comparison of the proportion of ART-conceived livebirths and maternal childbearing age across patients with aneuploid UPD-IDs

To investigate the effect of ART and maternal childbearing age for the development of aneuploid UPD-IDs in detail, we compared the proportion of ART-conceived live births and maternal childbearing age across patients with aneuploid UPD-IDs, focusing on SRS, KOS, and PWS, which are the three most frequent aneuploid UPD-IDs in the study. (Additional file 3: Fig. S2). The proportions of ART-conceived livebirths in patients with SRS, KOS, and PWS were 13.0%, 4.3%, and 12.7%, respectively, and were not significantly different from that in patients with other aneuploid UPD-IDs (13.8%). The median maternal childbearing age in patients with SRS, KOS, and PWS was not significantly different from that in patients with other aneuploid UPD-IDs. This result was consistent when we focused only on oUPD-IDs in each disease.

## Discussion

To our knowledge, this study is the first to evaluate the association of ART and parental age at childbirth for the development of aneuploid UPD-IDs, based on the robust data from a nationwide ART registry system. We included 130 patients with aneuploid UPD-IDs, the largest study sample to date, and compared them to the patients with epi-IDs, utilizing the results from our previous analyses focusing on epi-IDs [14]. Furthermore, we focused on the gametic origin of UPD based on the results of microsatellite analysis and SNP array analysis and conducted comparative analyses between aneuploid oocyte-mediated and sperm-mediated UPD-IDs, apparently for the first time.

Comparison between patients with aneuploid UPD-IDs and the general population or epi-IDs revealed two noteworthy findings. First, the proportion of ART-conceived livebirths in the patients with aneuploid UPD-IDs was generally comparable with that in the general population of maternal age ≥ 30 years. In addition, the frequency of ART-conceived livebirths in the patients with aneuploid UPD-IDs was lower than in epi-IDs. These results suggest that ART itself is not likely to facilitate UPD-IDs, and the effect of ART for the development of aneuploid UPD-IDs is lower than that for the development of epi-IDs. Second, the maternal childbearing age of the patients with aneuploid UPD-IDs was skewed toward the increased ages compared to that of the general population and epi-IDs. This result corresponds to the previous study showing older maternal childbearing age in patients with UPDmat compared to the non-UPD individuals [10]. Consistent with a previous study which argued against a positive association of ART with the development of UPD(15)mat [19], we suppose that advanced maternal childbearing age, not ART itself, facilitates the development of aneuploid UPD-IDs.

Several matters should be pointed out regarding comparison of the proportion of ART-conceived livebirths and parental age at childbirth between patients with aneuploid oocyte-mediated and sperm-mediated UPD-IDs. First, the proportion of ART-conceived livebirths in aneuploid oUPD-IDs was higher than that in sUPD-IDs, and most ART-conceived livebirths were identified in oUPD-IDs. In addition, maternal age at childbirth in patients with oUPD-IDs was higher than that in patients with sUPD-IDs. Based on the results of comparison between aneuploid UPD-IDs and the general population or epi-IDs, we consider that advanced maternal age leads to the large proportion of ART-conceived livebirths in patients with oUPD-IDs, and the ART procedure itself may not be associated with the development of oUPD-IDs. Our results reflect the fact that advanced maternal childbearing age increases meiotic disjunction, induces aneuploid oocytes, and then leads to UPD-IDs [7, 8]. Second, paternal age at childbirth in patients with oUPD-IDs was higher than that in patients with sUPD-IDs. Because maternal age and paternal age at childbirth were strongly correlated in our study, higher paternal age in aneuploid oUPD-IDs was due to the higher maternal age in this group. Therefore, we suggest that advanced paternal age does not likely contribute to the development of sperm aneuploidy leading to UPDs. Consistent with this, previous systematic reviews focusing on embryos derived from young oocyte donors concluded that advanced paternal age was not associated with aneuploidy rates [20, 21].

The comparison of the proportion of ART-conceived livebirths and maternal childbearing age across patients with aneuploid UPD-IDs revealed that both proportion of ART-conceived livebirths and maternal childbearing age were consistent in patients with SRS, KOS, PWS, and other aneuploid UPD-IDs. This finding suggests that SRS, KOS, and PWS are not particularly susceptible to the effects of ART or advanced maternal childbearing age.

Our study has some limitations. First, a large number of patients with SRS, KOS, and PWS were included in our study due to the characteristics of our laboratory. Regarding PWS, because Japanese health insurance covers FISH analysis of the 15q11-13 imprinted region for PWS patients, most PWS patients referred to our laboratory for a methylation analysis are those whose deletions have been ruled out by FISH analysis. Therefore, we detected a large number of patients with PWS caused by UPD(15)mat, the second most frequent etiology of PWS. Regarding KOS and SRS, our laboratory is the facility which conducts the largest number of genetic analyses of KOS and SRS in Japan. Therefore, we detected a large number of patients with KOS caused by UPD(14)pat, the most frequent etiology of KOS, and SRS caused by UPD(7)mat. Second, the confounding effect of infertility was not evaluated because we did not inquire about infertility in the questionnaire. Third, the effect of COS alone was not investigated. As in our previous report [14], we did not include COS into ART procedures based on the JSOG’s definition. Because previous studies revealed that COS induced oocyte aneuploidy [15, 16], further study is required to clarify the effects of COS on the development of UPD-IDs. Fourth, we determined UPD patterns and excluded mosaics with normal cell lineage based on the results of microsatellite analysis. When a meiotic error occurred without homologous recombination at meiosis 2 (M2), this might induce a disomic gamete consisting of identical chromatids which leads to full isodisomy through the TR or GC processes. Thus, it is possible that TR-type or GC-type UPD without recombination at M2 was incorrectly classified as MR-type UPD. In addition, we could not exclude cases with low mosaic rates in leukocytes. Because both MR-type UPD and PE-type UPD without recombination at mitosis shows full isodisomy, we could not distinguish MR-type UPD cases and PE-type UPD cases with low mosaic rates. Fifth, we classified GC-type UPDmat and UPDpat into aneuploid oocyte-mediated and sperm-mediated UPD, respectively. In fact, GC-type UPDmat is due to disomic oocytes and nullisomic sperm, and UPDpat is due to nullisomic oocytes and disomic sperm. This is an inevitable limitation of our study which distinguishes UPD pattern based on the results of microsatellite analysis. However, because GC-type UPD is extremely rare, the effect of this limitation is negligible.

## Conclusions

Different from the case of epi-IDs, ART itself is not likely to facilitate the development of aneuploid UPD-IDs. We concluded that advanced maternal age can be a risk factor for the development of aneuploid UPD-IDs, particularly oUPD-IDs.

## Methods

### Patients

We enrolled 130 patients with aneuploid UPD-IDs confirmed by molecular studies, including 77 previously reported patients (UPDmat of chromosome 7 (*n* = 9) [22], UPD(14)pat (*n* = 19) [23], UPD(14)mat (*n* = 17) [24], UPD(15)mat (*n* = 27) [19], and UPDmat of chromosome 20 (*n* = 5) [25]). We classified these 130 patients into UPD-IDs caused by aneuploid oocytes or sperm based on the UPD subtypes confirmed by microsatellite analysis and SNP array analysis. All patients were born from 1991 to 2017 and recruited from 2004 to 2019. We did not include the patients who were already identified with chromosomal structural abnormalities, such as ring chromosome or translocation. We obtained clinical information about parental age, conception (naturally or ART-conceived), and ART methods utilized in ART-conceived patients from the attending physicians by questionnaire. As in our previous report [14], we classified patients conceived with IVF, ICSI, and FET as ART-conceived livebirths based on the definition used in the JSOG database.

### Molecular studies

The flowchart of molecular studies is shown in Additional file 4: Fig. S3. To detect patients with UPD-IDs, we first conducted methylation analysis using pyrosequencing for nine IDs-related DMRs [22]. We excluded patients with abnormal methylation levels of either *H19/IGF2*:IG-DMR or *KCNQ1OT1*:TSS-DMR because these patients had suspected UPD of chromosome 11 which is only caused by PE, namely mosaic with normal cell lineage [1, 26]. When abnormally methylated DMR(s) other than *H19/IGF2*:IG-DMR and *KCNQ1OT1*:TSS-DMR were detected in patients, we conducted microsatellite marker analysis for the chromosomes including abnormally methylated DMR(s), i.e., chromosomes 6 [27], 7 [28], 14 [29], 15 [7], or 20 [25], using patients’ and their parental genomic DNA.

Primers utilized for pyrosequencing and microsatellite analyses are shown in Additional file 5: Table S2. Furthermore, when isodisomy was detected in patients, we conducted SNP array analysis with SurePrint G3 ISCA CGH + SNP Microarray Kit (Agilent Technologies, Santa Clara, CA, USA) and confirmed full isodisomy. Based on the results of microsatellite marker analysis and SNP array analysis, we determined UPD subtypes, such as TR, GC, MR, or PE (Additional file 1: Fig. S1). Then, we excluded patients with UPDs caused by PE which present with segmental isodisomy or mosaic with normal cell lineage, and consequently defined remaining UPDs as aneuploid UPDs. PE with a ring chromosome also results in full isodisomy through loss of a ring chromosome followed by duplication of a normal chromosome, but this type of UPD was not included in our study, because patients with chromosome abnormalities, including ring chromosomes, were excluded before conducting molecular analysis.

Aneuploid UPDs were classified into aneuploid oocyte-mediated or sperm-mediated UPDs based on the UPD subtypes. Maternal heterodisomy and paternal heterodisomy in one or more loci (TR or GC type) were classified into disomic oocyte-mediated matUPD and disomic sperm-mediated patUPD, respectively. Maternal full isodisomy and paternal full isodisomy detected by microsatellite and SNP array analysis (MR type) were classified into nullisomic sperm-mediated matUPD and nullisomic oocyte-mediated patUPD, respectively. Because a meiotic error without homologous recombination at M2 produces a disomic gamete consisting of identical chromatids that leads to full isodisomy, microsatellite analysis is not able to distinguish between MR-type UPD and TR-type or GC-type UPD without recombination at M2.

### Comparison of the proportion of ART-conceived livebirths and parental age

We compared (1) the proportion of ART-conceived livebirths and (2) maternal childbearing age in each year between patients with aneuploid UPD-IDs or epi-IDs and the general population. The birth data of the general population were obtained from the annual nationwide survey data from the Ministry of Health, Labor, and Welfare (http://www.mhlw.go.jp/toukei/list/81-1.html) and the registry data of JSOG (https://plaza.umin.ac.jp/~jsog-art/), as previously described [14]. Regarding the birth data of the patients with epi-IDs, we utilized the results of previous report [14]. In addition, we also compared the proportion of ART-conceived livebirths and parental age at childbirth between patients with oocyte-mediated and sperm-mediated UPD-IDs. Furthermore, we also compared the proportion of ART-conceived livebirths and maternal childbearing age between patients with SRS, KOS, or PWS and other aneuploid UPD-IDs.

### Statistical analysis

For the comparison of the distribution of maternal childbearing age between patients with aneuploid UPD-IDs or epi-IDs and the general population, we used the median and 2.5th and 97.5th percentiles for continuous variables as summary statistics. Statistical significance of the proportion of ART-conceived livebirths and parental age at childbirth was determined by Fisher’s exact test and Mann–Whitney’s *U*-test, respectively. The correlation between maternal age and paternal age was determined by Spearman’s rank-order test. Statistical analysis was performed by using R version 3.3.1. *P* < 0.05 was considered significant.

## Supplementary Information

**Additional file 1. Figure S1**. Schematic representation of the generation of uniparental disomy.

**Additional file 2. Table S1**. Molecular findings in the currently known imprinting disorders.

**Additional file 3. Figure S2**. Comparison of the proportion of ART-conceived livebirths and maternal childbearing age across patients with aneuploid UPD-IDs.

**Additional file 4. Figure S3**. Flowchart of molecular studies.

**Additional file 5. Table S2**. Primers utilized in this study.

## Data Availability

All data generated or analyzed during this study are available from the corresponding author on reasonable request.

## Abbreviations

ART: Assisted reproductive technology
AS: Angelman syndrome
BWS: Beckwith-Wiedemann syndrome
COS: Controlled ovarian stimulation
DMRs: Differentially methylated regions
epi-IDs: Epimutation-mediated imprinting disorders
FET: Frozen embryo transfer
GC: Gamete complementation
ICSI: Intracytoplasmic sperm injection
IDs: Imprinting disorders
IVF: In vitro fertilization
JSOG: Japanese society of obstetrics and gynecology
KOS: Kagami-Ogata syndrome
M1: Meiosis 1
M2: Meiosis 2
MR: Monosomy rescue
PE: Post-fertilization error
PWS: Prader-Willi syndrome
SRS: Silver-Russell syndrome
sUPD-IDs: Uniparental disomy-mediated imprinting disorders caused by aneuploid sperm
TR: Trisomy rescue
UPD: Uniparental disomy
UPD-IDs: Uniparental disomy-mediated imprinting disorders caused by aneuploid oocytes
UPD-IDs: Uniparental disomy-mediated imprinting disorders
UPD(mat): Maternal uniparental disomy
UPD(pat): Paternal uniparental disomy
UPD(7)mat: Maternal uniparental disomy of chromosome 7
UPD(14)mat: Maternal uniparental disomy of chromosome 14
UPD(14)pat: Paternal uniparental disomy of chromosome 15
UPD(15)mat: Maternal uniparental disomy of chromosome 15
