# Supplementary material for: Risk assessment of assisted reproductive technology and parental age at childbirth for the development of uniparental disomy-mediated imprinting disorders caused by aneuploid gametes
Source: Clin Epigenetics. 2023 May 6;15:78. doi: 10.1186/s13148-023-01494-w (PMC10163687; doi:10.1186/s13148-023-01494-w)
Supplement: Supplementary file 1 — Additional file 1. Figure S1. Schematic representation of the generation of uniparental disomy. [file 13148_2023_1494_MOESM1_ESM.pdf]

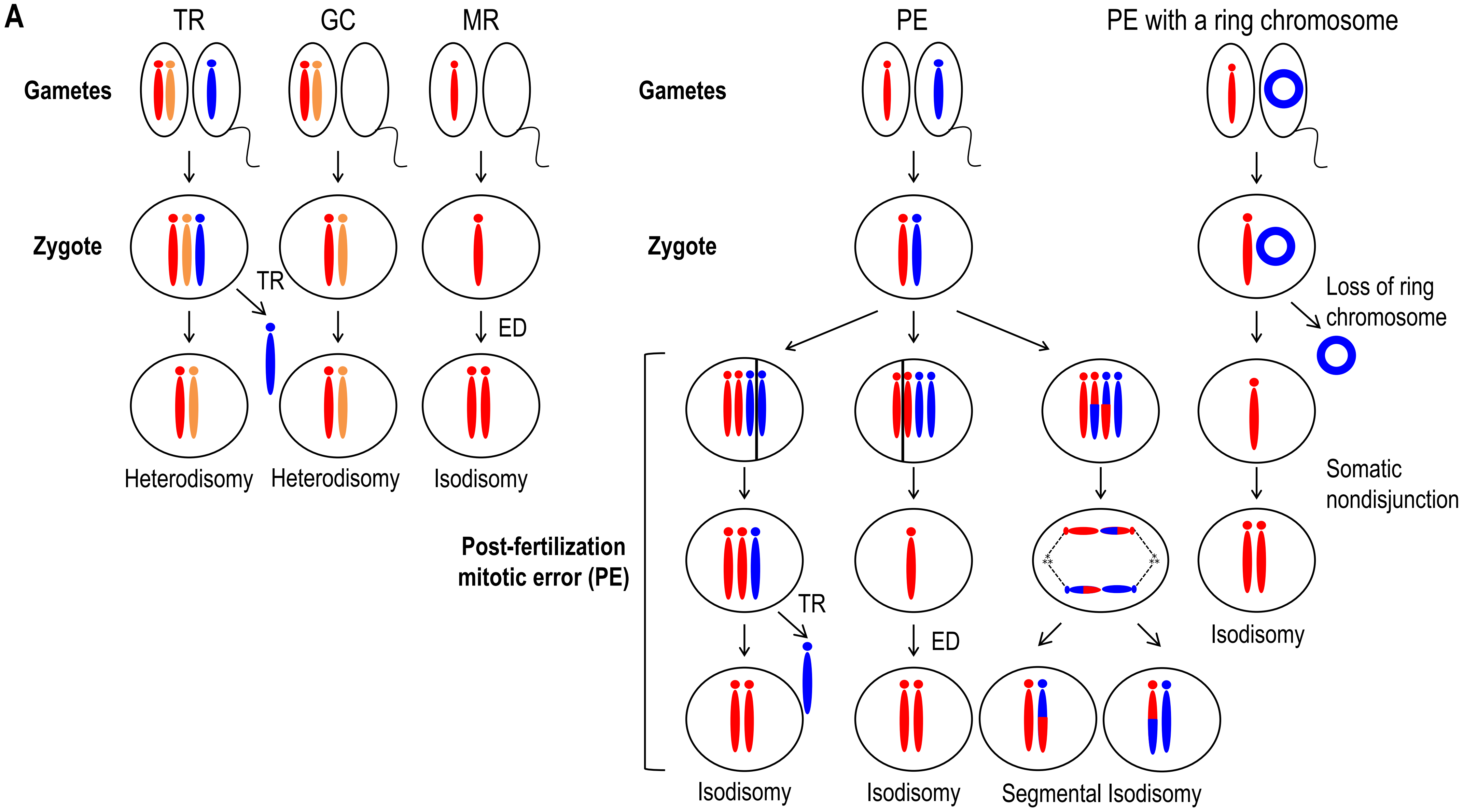

Figure S1. Schematic representation of the generation of uniparental disomy.

A. The generation of maternal uniparental disomy. TR following fertilization between a disomic oocyte and a normal sperm cell and GC caused by fertilization between a disomic oocyte and a nullisomic sperm cell result in maternal heterodisomy. MR following fertilization between a normal oocyte and a nullisomic sperm cell, PE without recombination, and PE with a ring chromosome result in maternal isodisomy. PE with recombination results in segmental maternal isodisomy. TR, trisomy rescue; MR, monosomy rescue; GC, gamete complementation; PE, post-fertilization mitotic error; ED, endoreduplication.

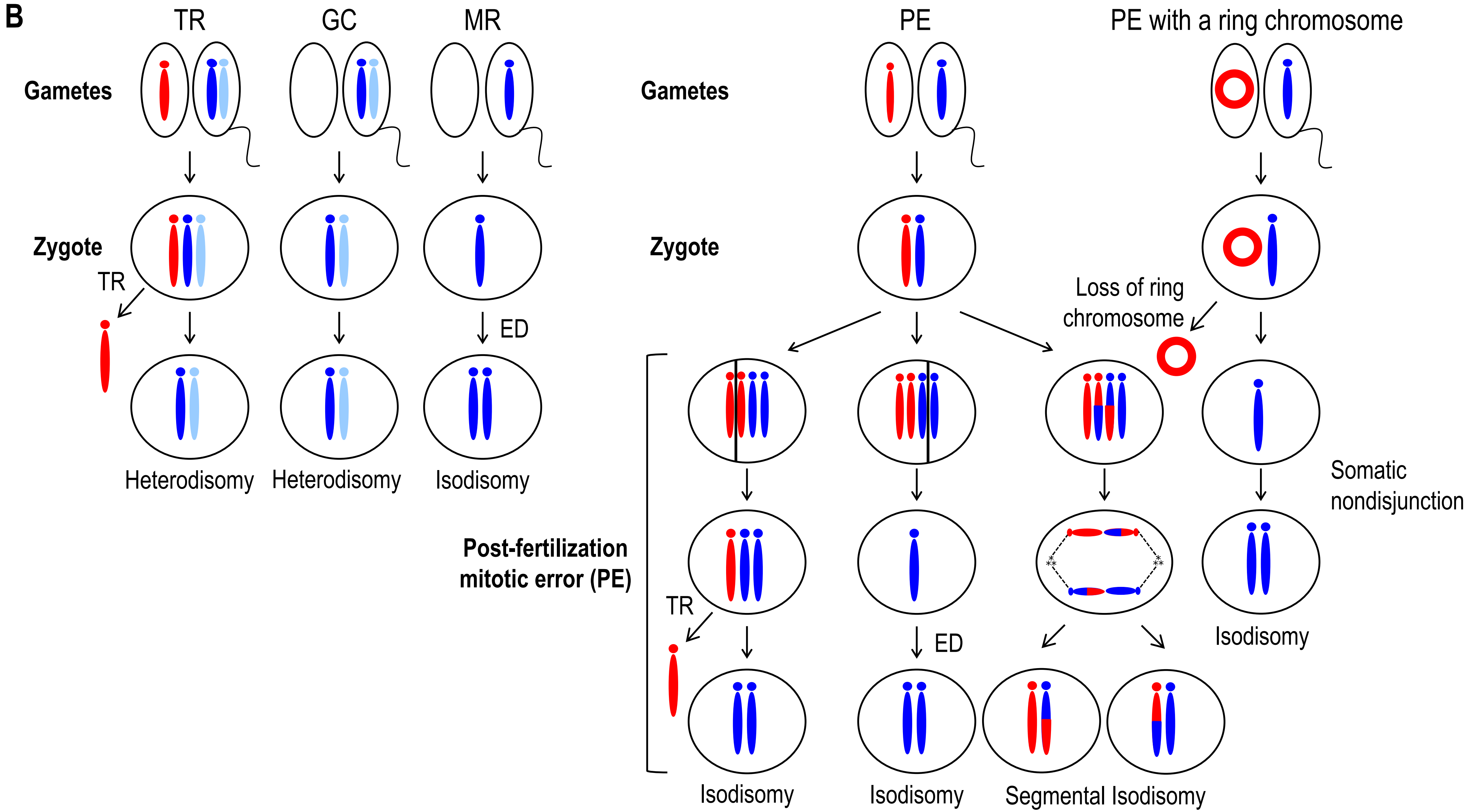

Figure S1. Schematic representation of the generation of uniparental disomy.

B. The generation of paternal uniparental disomy. TR following fertilization between a normal oocyte and a disomic sperm cell and GC caused by fertilization between a nullisomic oocyte and a disomic sperm cell lead to paternal heterodisomy. MR following fertilization between a nullisomic oocyte and a normal sperm cell, PE without recombination, and PE with a ring chromosome result in paternal isodisomy. PE with recombination results in segmental paternal isodisomy. TR, trisomy rescue; MR, monosomy rescue; GC, gamete complementation; PE, post-fertilization mitotic error; ED, endoreduplication.
