# Supplementary material for: Risk assessment of assisted reproductive technology and parental age at childbirth for the development of uniparental disomy-mediated imprinting disorders caused by aneuploid gametes
Source: Clin Epigenetics. 2023 May 6;15:78. doi: 10.1186/s13148-023-01494-w (PMC10163687; doi:10.1186/s13148-023-01494-w)
Supplement: Supplementary file 2 — Additional file 2. Table S1. Molecular findings in the currently known imprinting disorders. [file 13148_2023_1494_MOESM2_ESM.docx]

| **Supplementary Table S1.** Molecular findings in the currently known imprinting disorders | | | | | |  |  |
| --- | --- | --- | --- | --- | --- | --- | --- |
| Chromosome or imprinted region | Imprinting disorders associated with **maternal** UPD | Frequency | References |  | Imprinting disorders associated with **paternal** UPD | Frequency | References |
| 6q24 |  |  | 1 |  | **Transient neonatal diabetes mellitus** |  | 2 |
|  | UPD(6)mat | n = 14 |  |  | UPD(6)pat | 34.0% |  |
|  |  |  |  |  | duplication (6q24) pat | 25.2% |  |
|  |  |  |  |  | *PLAGL1*:alt-TSS-DMR hypomethylation | 32.0% |  |
| 7 | **Silver-Russell syndrome** |  | 2 |  |  |  | 3–7 |
|  | UPD(7)mat | 15.8% |  |  | UPD(7)pat | n = 5 |  |
| 11p15.5 | **Silver-Russell syndrome** |  | 2 |  | **Beckwith-Wiedemann syndrome** |  | 2 |
|  | UPD(11)mat* | 0.6% |  |  | UPD(11)pat* | 19.5% |  |
|  | 11p15 CNVs | 2.4% |  |  | 11p CNVs | 2.5% |  |
|  | *H19/IGF2*:IG-DMR hypomethylation | 67.6% |  |  | *H19/IGF2*:IG-DMR hypermethylation | 11.8% |  |
|  | unexpected molecular diagnosis | 13.6% |  |  | *KCNQ1OT1*:TSS-DMR hypomethylation | 64.0% |  |
|  |  |  |  |  | unexpected molecular diagnosis | 2.3% |  |
| 14q32.2 | **Temple syndrome** |  | 2 |  | **Kagami-Ogata syndrome** |  | 2 |
|  | UPD(14)mat | 48.8% |  |  | UPD(14)pat | 46.5% |  |
|  | deletion (14q32) pat | 11.0% |  |  | deletion (14q32) mat | 19.7% |  |
|  | *MEG3/DLK1*:IG-DMR *and MEG3*:TSS-DMR hypomethylation | 30.5% |  |  | *MEG3/DLK1*:IG-DMR *and MEG3*:TSS-DMR hypermethylation | 23.9% |  |
| 15q11-13 | **Prader-Willi syndrome** |  | 2 |  | **Angelman syndrome** |  | 2 |
|  | UPD(15)mat | 24.7% |  |  | UPD(15)pat | 9.3% |  |
|  | deletion (15q11–q13) pat | 39.4% |  |  | deletion (15q11–q13) mat | 39.3% |  |
|  | duplication (15q11–q13) mat | 10.3% |  |  | duplication (15q11–q13) pat | 1.5% |  |
|  | *SNURF*:TSS-DMR hypermethylation | 10.0% |  |  | *SNURF*:TSS-DMR hypomethylation | 28.5% |  |
|  |  |  |  |  | *UBE3A* gene mutations | 15.7% |  |
| 20q13.3 |  |  | 8 |  | **PHP and related disorders** |  | 9 |
|  | UPD(20)mat | n = 24 |  |  | UPD(20)pat | 2.7% |  |
|  |  |  |  |  | *GNAS* gene mutations | 37.7% |  |
|  |  |  |  |  | broad *GNAS* methylation defects | 38.0% |  |
|  |  |  |  |  | *STX16* deletion | 13.5% |  |
|  |  |  |  |  | Structural rearrangement | 2.4% |  |

* Almost all UPD(11)mat patients and UPD(11)pat patients were mosaic with normal cell linage.

UPD, uniparental disomy; DMR, differentially methylated region; PHP, pseudohypoparathyroidism; pat, paternal; mat, maternal; CNVs, copy number variants; UPD(6)mat, maternal uniparental disomy of chromosome 6; UPD(6)pat, paternal uniparental disomy of chromosome 6; UPD(7)mat, maternal uniparental disomy of chromosome 7; UPD(7)pat, paternal uniparental disomy of chromosome 7; UPD(11)mat, maternal uniparental disomy of chromosome 11; UPD(11)pat, paternal uniparental disomy of chromosome 11; UPD(14)mat, maternal uniparental disomy of chromosome 14; UPD(14)pat, paternal uniparental disomy of chromosome 14; UPD(15)mat, maternal uniparental disomy of chromosome 15; UPD(15)pat, paternal uniparental disomy of chromosome 15; UPD(20)mat, maternal uniparental disomy of chromosome 20; UPD(20)pat, paternal uniparental disomy of chromosome 20.

References

1. Lazier J, Martin N, Stavropoulos JD, Chitayat D.Maternal uniparental disomy for chromosome 6 in a patient with IUGR, ambiguous genitalia, and persistent mullerian structures. Am J Med Genet A. 2016;170:3227–30.
2. Mackay D, Bliek J, Kagami M, Tenorio-Castano J, Pereda A, Brioude F, et al. First step towards a consensus strategy for multi-locus diagnostic testing of imprinting disorders. Clin Epigenetics. 2022;14:143.
3. Höglund P, Holmberg C, de la Chapelle A, Kere J. Paternal isodisomy for chromosome 7 is compatible with normal growth and development in a patient with congenital chloride diarrhea. Am J Hum Genet. 1994;55:747–52.
4. Pan Y, McCaskill CD, Thompson KH, Hicks J, Casey B, Shaffer LG, et al. Paternal isodisomy of chromosome 7 associated with complete situs inversus and immotile cilia. Am J Hum Genet. 1998;62:1551–5.
5. Fares F, David M, Lerner A, Diukman R, Lerer I, Abeliovich D, et al. Paternal isodisomy of chromosome 7 with cystic fibrosis and overgrowth. Am J Med Genet A. 2006;140:1785–8.
6. Le Caignec C, Isidor B, de Pontbriand U, David V, Audrezet MP, Ferec C, et al. Third case of paternal isodisomy for chromosome 7 with cystic fibrosis: a new patient presenting with normal growth. Am J Med Genet A. 2007;143a:2696–9.
7. Nakamura A, Muroya K, Ogata-Kawata H, Nakabayashi K, Matsubara K, Ogata T, et al. A case of paternal uniparental isodisomy for chromosome 7 associated with overgrowth. J Med Genet. 2018;55:567–70.
8. Tannorella P, Minervino D, Guzzetti S, Vimercati A, Calzari L, Patti G, et al. Maternal Uniparental Disomy of Chromosome 20 (UPD(20)mat) as Differential Diagnosis of Silver Russell Syndrome: Identification of Three New Cases. Genes (Basel). 2021;12:588.
9. Elli FM, Linglart A, Garin I, de Sanctis L, Bordogna P, Grybek V, et al. The Prevalence of GNAS Deficiency-Related Diseases in a Large Cohort of Patients Characterized by the EuroPHP Network. J Clin Endocrinol Metab. 2016;101:3657–68.
