# Supplementary material for: Risk assessment of assisted reproductive technology and parental age at childbirth for the development of uniparental disomy-mediated imprinting disorders caused by aneuploid gametes
Source: Clin Epigenetics. 2023 May 6;15:78. doi: 10.1186/s13148-023-01494-w (PMC10163687; doi:10.1186/s13148-023-01494-w)
Supplement: Supplementary file 3 — Additional file 3. Figure S2. Comparison of the proportion of ART-conceived livebirths and maternal childbearing age across patients with aneuploid UPD-IDs. [file 13148_2023_1494_MOESM3_ESM.pdf]

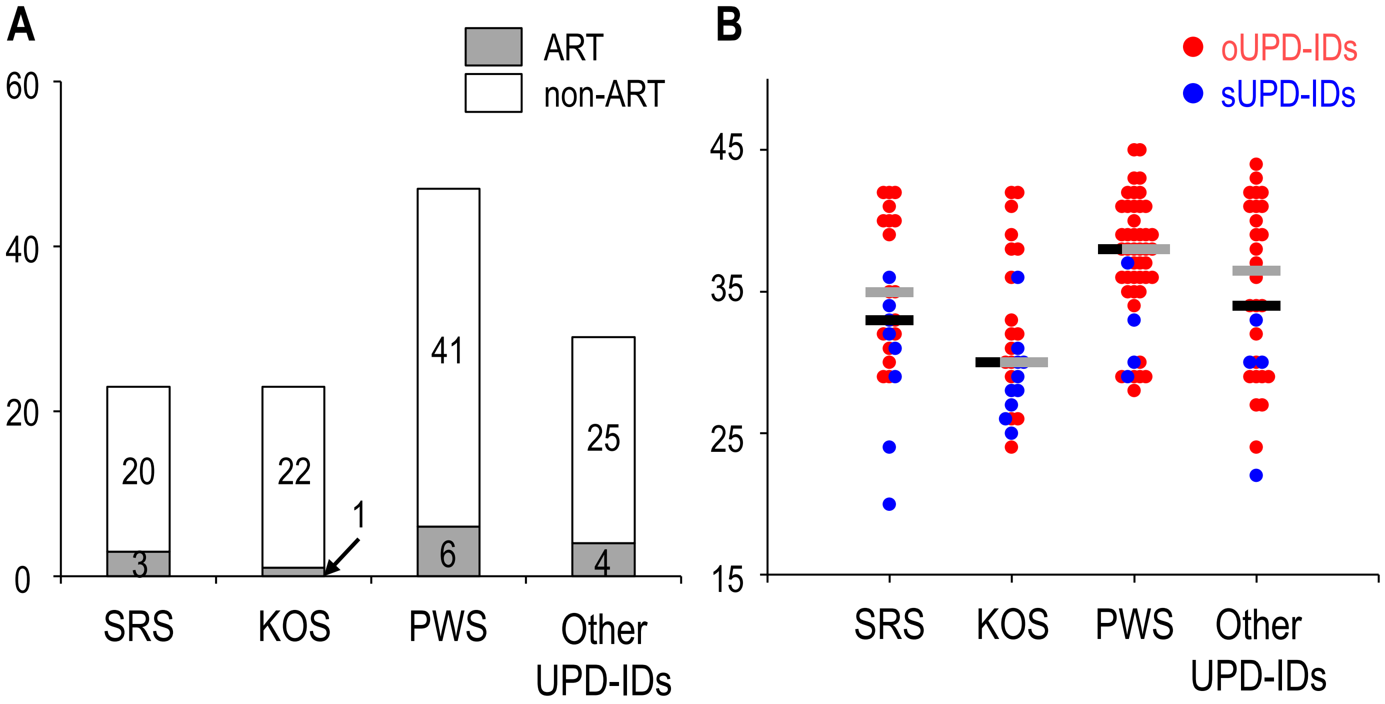

Figure S2. Comparison of the proportion of ART-conceived livebirths and maternal childbearing age across patients with aneuploid UPD-IDs.

A. The proportion of ART-conceived livebirths across patients with aneuploid UPD-IDs. Gray and white bars indicate the number of ART-conceived and non-ART-conceived livebirths, respectively. **B.** The distribution of maternal childbearing age across patients with aneuploid UPD-IDs. Red and blue dots indicate the maternal age of the patients with oUPD-IDs and sUPD-IDs, respectively, in each group. Black and gray bars indicate the median maternal age of the patients with all aneuploid UPD-IDs and oUPD-IDs, respectively, in each group. ART, assisted reproductive technology; UPD-IDs, uniparental disomy-mediated imprinting disorders; oUPD-IDs, uniparental disomy-mediated imprinting disorders caused by aneuploid oocytes; sUPD-IDs, uniparental disomy-mediated imprinting disorders caused by aneuploid sperm; SRS, Silver-Russell syndrome; KOS, Kagami-Ogata syndrome; PWS, Prader-Willi syndrome; IDs, imprinting disorders.
