# Supplementary material for: Risk assessment of assisted reproductive technology and parental age at childbirth for the development of uniparental disomy-mediated imprinting disorders caused by aneuploid gametes
Source: Clin Epigenetics. 2023 May 6;15:78. doi: 10.1186/s13148-023-01494-w (PMC10163687; doi:10.1186/s13148-023-01494-w)
Supplement: Supplementary file 4 — Additional file 4. Figure S3. Flowchart of molecular studies. [file 13148_2023_1494_MOESM4_ESM.pdf]

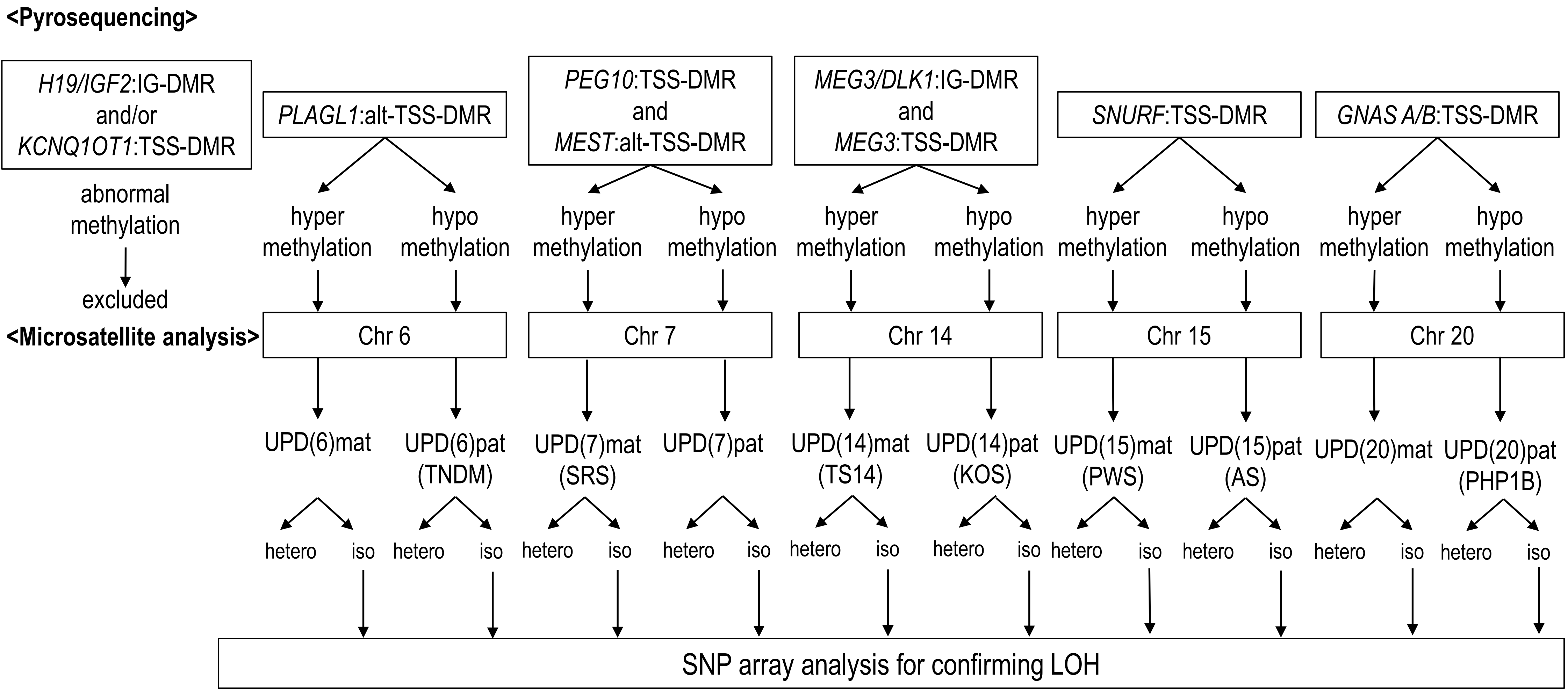

Figure S3. Flowchart of molecular studies. DMR, differentially methylated region; Chr, chromosome; UPD(6)mat, maternal uniparental disomy of chromosome 6; UPD(6)pat, paternal uniparental disomy of chromosome 6; UPD(7)mat, maternal uniparental disomy of chromosome 7; UPD(7)pat, paternal uniparental disomy of chromosome 7; UPD(14)mat, maternal uniparental disomy of chromosome 14; UPD(14)pat, paternal uniparental disomy of chromosome 14; UPD(15)mat, maternal uniparental disomy of chromosome 15; UPD(15)pat, paternal uniparental disomy of chromosome 15; UPD(20)mat, maternal uniparental disomy of chromosome 20; UPD(20)pat, paternal uniparental disomy of chromosome 20; TNDM, transient neonatal diabetes mellitus; SRS, Silver-Russell syndrome; TS14, Temple syndrome; KOS, Kagami-Ogata syndrome; PWS, Prader-Willi syndrome; AS, Angelman syndrome; PHP1B, pseudohypoparathyroidism 1B; hetero, heterodisomy; iso, isodisomy; LOH, loss of heterozygosity.
