# Supplementary material for: Risk assessment of assisted reproductive technology and parental age at childbirth for the development of uniparental disomy-mediated imprinting disorders caused by aneuploid gametes
Source: Clin Epigenetics. 2023 May 6;15:78. doi: 10.1186/s13148-023-01494-w (PMC10163687; doi:10.1186/s13148-023-01494-w)
Supplement: Supplementary file 5 — Additional file 5. Table S2. Primers utilized in this study. [file 13148_2023_1494_MOESM5_ESM.docx]

| **Supplementary Table S2.** Primers utilized in this study. | |  |  |  |  |
| --- | --- | --- | --- | --- | --- |
| <Pyrosequencing analysis> | Forward (5’ → 3’) | Reverse (5’ → 3’) | Sequence primer | AT | PS |
| *PLAGL1*:alt-TSS-DMR | GGGGTAGTYGTGTTTATAGTTTAG | biotin-CCCAAACACCTACCCTAC | GGGTAGTYGTGTTTATAGTTTAGT | 55 | 146 |
| (6q24.2) | chr6: 144329336-144329359 | chr6: 144329214-144329231 | chr6: 144329335-144329358 |  |  |
| *PEG10*:TSS-DMR | AGAAATTTGATTGYGTTTTGAGGAGAAT | ACCTTTAAAACTTAATTTCCCCATTTAT | AGTTTGGYGAAAGGTT | 55 | 344 |
| (7q21.3) | chr7: 94285716-94285743 | chr7: 94286028-94286059 | chr7: 94285762-94285777 |  |  |
| *MEST*:alt-DMR | GTGTGGTTGGYGGTTTTGGGATTA | biotin-ACACCCCCTCCTCAAATA | TGTTTTTGGGYGAAAATTTTAT | 55 | 143 |
| (7q32.2) | chr7: 130132206-130132229 | chr7: 130132332-130132348 | chr7: 130132276-130132297 |  |  |
| *MEG3/DLK1*:IG-DMR | ATTTGGTATTTGTAGTTTTATGTTAAGATG | biotin-AATCAAAACAACTCAAATCCTTTATAAC | AATTGGGTTTGTTAGTAG | 54 | 164 |
| (14q32.2) | chr14: 101275613-101275642 | chr14: 101275749-101275776 | chr14: 101275685-101275702 |  |  |
| *MEG3:*TSS-DMR | TTGTGTTTGAATTTATTTTGTTT | biotin-CCCCAAATTCTATAACAAATTACTCT | GTGTTTGAATTTATTTTGTTT | 54 | 167 |
| (14q32.2) | chr14: 101292170-101292192 | chr14: 101292311-101292336 | chr14: 101292172-101292192 |  |  |
| *SNURF*:TSS-DMR | GGGATATTTGAGATTTTGAAAGAA | biotin-AATACAAAACCTCCCCTACT | GTTATTTTTTTTATTTGGGAGGA | 54 | 110 |
| (15q11.2) | chr15:25200788-25200813 | chr15:25200823-25200837 | chr15:25200880-25200897 |  |  |
| *GNAS A/B*:TSS-DMR | GGGATATTTGAGATTTTGAAAGAA | biotin-AATACAAAACCTCCCCTACT | GTTATTTTTTTTATTTGGGAGGA | 52 | 217 |
| (20q13.32) | chr20:57463531-57463555 | chr20:57463727-57463746 | chr20:57463630-57463652 |  |  |

| <Microsatellite analysis> | Forward (5’ → 3’) | Reverse (5’ → 3’) | AT | PS |
| --- | --- | --- | --- | --- |
| *D6S1574* | AAGAACTTCCCAAACCAAT | AACCATCCAGGACATCAA | 57 | ~184 |
| *D6S1610* | CCTGGTGAGATAGATGCTTG | ATTTCCAGCAGAGCCTTG | 57 | ~143 |
| *D6S257* | GAGAACTCGTCCTTTGGTCC | TGAGAAAATGTTCAGGCTAAAGATA | 57 | ~186 |
| *D6S1671* | TTTGGTCAATTTCAATCTGTAG | ATCCTCCAGGGGTGCT | 57 | ~290 |
| *D6S434* | CAGGTAGTCCCCCAAAATCA | AGCTCAGGGCTTATGCCAGT | 57 | ~234 |
| *D6S287* | ATATTAGTGCCTTATGCTTCTG | AAATTGGATATTCATGCTTG | 57 | ~171 |
| *D6S1654* | ATTTGCCGCTTTCTATGG | TGTATGTTCAGTTACTGGACAGG | 57 | ~280 |
| *D6S1599* | TGTTTTCCACAGGTTCCAG | CTTCAGATGTAGGCTCCACG | 57 | ~155 |
| *D6S1657* | AAAGATACAGGCCCACAT | ACCATTTTGCCATCGT | 57 | ~221 |
| *D7S531* | AAACTGTGGTCCTGGCTG | AAACTAGAGTCCTGGCCTGA | 58 | ~233 |
| *D7S507* | GATTGCTTAATACCTTCC | ACCAAGAATTTATCCCAC | 60 | ~152 |
| *D7S2552* | TCTAGGGTGGGTTTGC | TGTTCACTGGATCTCATTC | 60 | ~232 |
| *D7S2429* | CAGTGCTGGAGTTGTTCAAG | CTGGGAGTCAAGTGTTTTGG | 60 | ~173 |
| *D7S2504* | TGTGGTACAATTTCAGACACATAA | CTGGAAACCAGTGTTTTCACTT | 60 | ~197 |
| *D7S672* | CTACCAGTAGCCTCTTCAAGTG | TACTTAGAGGACCCACTTTGG | 57 | ~139 |
| *D7S500* | CTGTGTTTTAAAAGCCACCA | TGATTGAGGAACTGAACTTACC | 60 | ~188 |
| *D7S2442* | TGAGCCAAGATCACAGCACT | CTGGAAGCAACAGATGTCACTA | 60 | ~226 |
| *D7S2465* | CCACATAAGCATAAACTC | TTCTCCAGCATTTTTGGTC | 60 | ~181 |
| *D14S608* | TAAAGGTTTATCCATGCTGTAGC | ACGTGGTACAGGTAGATAAATGG | 57 | ~224 |
| *D14S588* | GCCGAAAGAAAGAAAAAAGG | CGAATGCATACTTGCTGTTG | 57 | ~141 |
| *D14S617* | TTTTAGGTGGCCACCATCTA | CCAGTTTAGGCAACAGAACA | 57 | ~173 |
| *D14S267* | TTAATGCCCACTGAATGCT | AAGGCAGCCCTGGTTT | 57 | ~225 |
| *D14S250* | GAAACTGGAACCACTGTGC | ACCCCTGCATTGTTTGAG | 57 | ~179 |
| *D14S1006* | TTCCACAGGGCAAGCAGTA | TTCTGGCAAAACCCAACC | 57 | ~155 |
| *D14S985* | CAGTGTGACCTTAAACAAGTCG | CCTGTGGGGTAGATACACGA | 57 | ~142 |
| *D14S292* | CTGTGTGGTGCATCAATG | CATGAAGGCAGCCTCA | 57 | ~133 |
| *D15S541* | GCATTTTTGGTTACCTGTATG | GTCTTCCAGGTTTATGGTTGTC | 57 | ~150 |
| *D15S542* | AGCAGACTCCGGAACCTCATC | CCTGCCTTCTTGCTGGGGCTG | 57 | ~140 |
| *D15S1035* | CACCCCCATGCAGAGT | AAGGCCAAGACCTGCC | 57 | ~262 |
| *D15S128* | GCTGTGTGTAAGTGTGTTTTATATC | GCAAGCCAGTGGAGAG | 57 | ~209 |
| *D15S1007* | AGCTCCTATATGTCTTCACACAG | CTCCATTCCCATACGTCC | 57 | ~189 |
| *D15S117* | GCACCAACAACTTATCCCAA | CCCTAAGGGGTCTCTGAAGA | 57 | ~150 |
| *D15S205* | CTTAATGGTTTGGCAGGATA | AGCTTAAAANCAAAATCTCCC | 57 | ~170 |
| *D15S127* | CCAACCACACTGGGAA | AACAGTTGCCCACGGT | 57 | ~147 |
| *D15S642* | CAGTTACCCAGGAAGCTGAA | AGATGCCGCCTGTACTAATG | 57 | ~218 |
| *D20S117* | GCACAAAGCAGACAGGAATA | CCAAGTTTGCAAGTAAGTAAGG | 57 | ~129 |
| *D20S889* | GGTTTGGTGGAATCCTCTC | CATCTTTCAAATGGGATAATGG | 57 | ~298 |
| *D20S192* | TGCTGTGTAGAAAACTTGAACTGA | ACAGTCATCCCCAAAATCCC | 57 | ~299 |
| *D20S917* | GATTTTGGTCTACGGTTTCCTTATT | ATCATTTGGGAAGTTTCCATT | 57 | ~167 |
| *D20S186* | GCACAAAGCAGACAGGAATA | CCAAGTTTGCAAGTAAGTAAGG | 57 | ~126 |
| *D20S112* | ATGGGTGTGCCAAATCTC | TTCTTGTAAGTCAGACAGCATCA | 57 | ~221 |
| *D20S912* | CGGCTCTAGCATTCATTTG | GGCTACTTAGACTAANTCATGGACC | 57 | ~283 |
| *D20S195* | GTACCTCCTCCAGGCTTC | AGGGGTGTATGTGTGCAT | 57 | ~261 |
| *D20S107* | CTACATGATGCCTCTTGGGA | TCAGACAATGGCAAATTCCT | 57 | ~278 |
| *D20S119* | CTGACACAGTTTCAGTATCTCTATC | TTTCCAGATTTAGGGGTGTATG | 57 | ~118 |
| *D20S120* | TTTTACTAAGGAGACTCAACAGGG | TTGCACAATGCCTGGAA | 57 | ~241 |
| *D20S100* | ATTGGGTTTACTTGTGCCTT | CGTGATTTCATTTCTTGCTG | 57 | ~218 |

chr, chromosome; AT, annealing temperature (°C); PS, product size (bp). Y: C or T (pyrimidine).

Physical positions of the primers are based on the NCBI database (Genome Build 37.1).
